# Circulating microRNAs as Biomarkers, Therapeutic Targets, and Signaling Molecules

**DOI:** 10.3390/s120303359

**Published:** 2012-03-08

**Authors:** Seena K. Ajit

**Affiliations:** Pharmacology and Physiology, Drexel University College of Medicine, Mail Stop 488, 245 North 15th Street, Philadelphia, PA 19102, USA; E-Mail: seena.ajit@drexelmed.edu; Tel.: +1-215-762-2218; Fax: +1-215-762-2299

**Keywords:** microRNA, biomarker, exosome

## Abstract

Small noncoding microRNAs (miRNAs) are important regulators of post-transcriptional gene regulation and have altered the prevailing view of a linear relationship between gene and protein expression. Aberrant miRNA expression is an emerging theme for a wide variety of diseases, highlighting the fundamental role played by miRNAs in both physiological and pathological states. The identification of stable miRNAs in bodily fluids paved the way for their use as novel biomarkers amenable to clinical diagnosis in translational medicine. Identification of miRNAs in exosomes that are functional upon delivery to the recipient cells has highlighted a novel method of intercellular communication. Delivery of miRNAs to recipient cells via blood, with functional gene regulatory consequences, opens up novel avenues for target intervention. Exosomes thus offer a novel strategy for delivering drugs or RNA therapeutic agents. Though much work lies ahead, circulating miRNAs are unequivocally ushering in a new era of novel biomarker discovery, intercellular communication mechanisms, and therapeutic intervention strategies.

## 1. Introduction

MicroRNAs (miRNAs) are small noncoding RNA molecules that can modulate gene expression in a wide range of biological process. They regulate gene expression by binding to the target messenger RNA (mRNA). Seed sequence complementarity of ∼7 base pairs enables miRNA to bind to the target mRNA. The direct interaction of the seed sequence with their target mRNAs can result in the inhibition of translation or in the reduction in the stability of the mRNA, both of which can result in decreased expression of the target protein [1]. Most mRNA targets contain multiple miRNA binding sites, and each miRNA can regulate multiple genes. The discovery of miRNAs has revolutionized cell biology and has permanently altered the prevailing view of a linear relationship between gene and protein expression. Considering the fundamental role miRNAs play in mediating biological events, it is not surprising that any perturbations in the homeostasis result in alterations of the miRNA expression profile [2–6]. Overexpression, deletion, alterations in epigenetic regulation, mutations in the sequence of the mature miRNA resulting in elimination of or changes in binding affinity to target mRNAs, or mutations in target mRNAs are all possible mechanisms resulting in altered expression and, in many cases, in disease. These changes are thus a reflection of imbalance in the regulatory network and have tremendously enhanced our understanding of gene regulation, in the context of both normal and disease states. Though the mechanistic basis of alterations in miRNA, especially if the changes observed are a cause or consequence of a malfunction in the cell, is not well understood, their identification offers a plethora of information resulting in the identification of biomarkers. In addition, miRNAs are being explored for therapeutic intervention as direct targets. The last update (Version 18) of the miRBase database (http://mirbase.org/), the online repository for published miRNA sequences, reported 18,226 entries representing hairpin precursor miRNAs, expressing 21,643 mature miRNA products, in 168 species. The number reported for *Homo sapiens* is 1,527. The current version of miR2Disease, a manually curated database for miRNA deregulation in humans diseases, documents 3,273 entries linking 349 human miRNAs and 163 diseases (http://www.mir2disease.org/) [7].

## 2. Circulating miRNAs

Recent identification of stable miRNAs in bodily fluids [8–11] paved the way for their use as novel biomarkers amenable to clinical diagnosis in translational medicine. The simplicity of miRNA detection, combined with the observed specificity, has many researchers predicting a revolution in the discovery of biomarkers [12]. Secreted miRNAs have many requisite features of good biomarkers. Whereas proteins are more diverse and therefore potentially more informative, the complex composition of protein in blood, post-translational modifications, low relative abundance, sequence variations, and difficulties associated with the development of high-affinity detection agents render the discovery and development of new protein-based biomarkers challenging and expensive. In addition to their stability in various bodily fluids, secreted miRNAs offer additional advantages. Most miRNA sequences are conserved across species; the expression of some miRNAs is specific to tissues or biological stages; and the level of miRNAs can be easily measured by quantitative PCR, which allows for high-precision signal amplification. Thus, detection of miRNA can be sensitive, predictive, specific, robust, translatable, and noninvasive, all characteristics of the ideal biomarker [13]. A number of methods are currently available for the detection and quantification of miRNAs. These methods have recently been reviewed in detail, and the advantages and disadvantages of each technique have been discussed [14]. The advent of the next generation sequencing technologies has greatly enhanced discovery of novel miRNAs.

Several factors pose potential problems for the successful application of circulating miRNAs as biomarkers. One major issue is the lack of standardized procedures that can introduce bias in the interpretation of results. Variability, which makes cross comparison of studies published from different laboratories difficult, can be due to a number of factors. Differences in sample collection, storage, RNA isolation, accurate assessment of quantity and quality of miRNA, and the preamplification step when using small quantities of starting material can all be contributing factors.

Microarrays, quantitative real-time PCR, and next-generation sequencing are the three platforms generally used in miRNA quantification. Several methods are used in data normalization to account for variability, including mean, quantile, endogenous, and discovered miRNAs [15–18]. To calculate the mean normalization value, the average of all miRNAs is subtracted from each cycle threshold value. Quantile normalization uses the ranking of the raw cycle threshold expression values on each plate. Endogenous control miRNAs are usually recommended by vendors. Alternatively, one can identify endogenous controls appropriate for the current study using various stability criteria. Identification of miRNAs that are minimally perturbed and most stable across all samples in a study can thus serve as endogenous controls. It has been observed that miRNAs recommended as controls were altered significantly in certain pathological states [19]. miRNA control for tissues may not be appropriate for body fluids and *vice versa* in both normal and diseased states. Thus it is unlikely that a set of reference or housekeeping miRNAs with universal applicability will be identified. However, a series of guidelines regarding miRNA isolation, quantification, and normalization for different platforms can lead to meaningful comparison of findings from different studies.

We have observed that miRNA profiles in control samples have some variability [20]. The underlying assumption in every study involving patient populations is that control individuals are healthy. Considering the fundamental role played by miRNAs in cellular function, it is conceivable that any fluctuations, either epigenetically induced or other factors such as ethnicity, age, gender, and diurnal changes can influence miRNA expression levels. Thus it is important to try to increase the cohort sizes of both patients and control samples to obtain reproducible results. However, this goal may not be always possible due to difficulty in sample procurement and the cost associated with these types of studies.

It is also becoming increasingly evident that some miRNAs can be deregulated in multiple disorders or in different types of cancer [21,22]. This observation is not surprising, considering the facts that each miRNA can bind multiple mRNA targets and that often there may be an overlap in the signal transduction cascade. Though changes in miRNA expression have been widely reported, whether these modulations are the cause or the consequence of malfunction is mostly unknown. Identification of multiple miRNA changes is extremely valuable because specific signatures of miRNA combinations unique to a normal physiological or pathological state can serve as a useful reference. In addition, assigning rank order of biological relevance can help address challenges associated with identification of same miRNAs in multiple disease states.

Much progress has been made in oncology where miRNAs have been associated with disease progression, clinical outcome, recurrence, and metastasis [21,23–25]. Studies of miRNAs encoded by pathogenic human viruses suggest that viral miRNAs can regulate host genes [26]. Differences in viral and host miRNAs can be used to develop diagnostics indicative of viral infections. In addition to major disease areas, miRNA expression profiles have been used in a number of areas that impact human health. Expression profiles of miRNAs in transplant recipients indicate their utility as biomarkers in determining allograft status by predicting the individual risk of rejection based on the immune response [27]. Another application of miRNA biology is in prenatal diagnostics because miRNAs expressed in the placenta can be detected in the maternal plasma. Conventional prenatal diagnostic methods such as chorionic villus sampling and amniocentesis are invasive and risky. Alterations in the miRNA profile at various stages of pregnancy, including the postnatal period, suggest that miRNAs could be used as an indicator to monitor the physiological state and could serve as a noninvasive prenatal diagnostic tool [28]. Yet another use of circulating miRNA is in determining the quality of stored blood. Managing the supply of transfusion-quality blood revolutionized health care; recent studies have shown that changes in miRNAs during storage may be indicative of the quality of the stored blood [29].

Anucleate blood cells such as platelets and erythrocytes were considered relatively inert due to the lack of a nucleus and *de novo* transcription. However, recent studies have demonstrated the presence of miRNAs and functional miRNA pathways in addition to mRNAs in human platelets [30]. The detection of extracellular miRNAs in serum thus raised the question of the stability of miRNAs. It has been suggested that miRNAs must be protected by a lipoprotein complex in the serum. It was demonstrated that exosomes, which are membrane vesicles released by cells, contained both mRNA and miRNA that were functional when delivered to another cell [31]. This finding revealed a new mechanism of miRNA-mediated cell-cell communication and signaling through exosomes and is discussed below.

## 3. Exosome-Mediated miRNA Transfer in Intercellular Communication

Exosomes are small 40- to 100-nm membrane vesicles that are released by different types of cells. They can be found in various bodily fluids including plasma, urine, amniotic fluid, and saliva. Exosomes are formed from endosomes, which in turn arise from an inward budding of the plasma membrane into the cytoplasm. Inward budding of the endosomal membrane gives rise to cytosolic multivesicular bodies (MVB). When MVB fuse with the plasma membrane, exosomes are released [32,33]. The presence of mRNA and miRNA in exosomes from mouse and human mast cell lines was reported by Valadi and coworkers [31]. Exosomes contain proteins, miRNAs, and mRNAs, and the exosomal lipid bilayer protects the genetic information from degradation. The Web site http://exocarta.org/ is a manually curated database of exosomal proteins, RNAs, and lipids identified in exosomes from multiple organisms [34,35]. Version 3.1 of ExoCarta contains information on 11,261 proteins, 2,375 mRNAs, and 764 miRNA entries obtained from 134 exosomal studies. Exosomes have a number of biological functions, including immune response, antigen presentation, intracellular communication, and the transfer of RNA and proteins. A number of excellent reviews have addressed the topic in depth [36–39].

A recent study showed that extracellular miRNAs exist predominantly free of exosomes/microvesicles and are associated with argonaute (Ago) proteins. Ago proteins can bind directly to mature miRNAs, and it is in combination with Ago proteins that a single miRNA targets hundreds of different mRNAs. Though the authors do not reject the possibility that some miRNAs can be associated with exosomes, they hypothesize that extracellular miRNAs are predominantly by-products of dead cells that remain in the extracellular space because of the highly stable argonaute2 (Ago2) protein and the Ago2-miRNA complex [40].

Intercellular communication was thought to be limited to cell-to-cell adhesion conduits (gap junctions) or secreted signals such as hormones, neurotransmitters, and cytokines released from cells and acting in an autocrine or paracrine manner. It has been shown that miRNAs are transported in plasma and delivered to recipient cells by high-density lipoproteins, resulting in modulation of target mRNAs [41]. Exosomes, on fusion with the plasma membrane of the recipient cell, transfer their internal components to the target cell. Delivery of miRNAs to recipient cells via blood, with functional gene regulatory consequences, opens up novel avenues for target intervention. In fact, exosomes offer a novel strategy for delivering cargos of drugs or RNA therapeutic agents. If the exosomes are derived from the same patient and reintroduced after loading with the molecule of interest, they should be better tolerated by the immune system. This approach may hasten the development of personalized medicine and therapy in the clinic.

## 4. Therapeutic Intervention Strategies

It is now widely established in oncology that miRNAs can function as oncomirs or tumor suppressors, depending on the genes they regulate and their cellular context. Different strategies for overexpression or downregulation of specific miRNAs are being pursued for therapeutic intervention for various diseases. miRNA-based therapy can be described as a double-edged sword. On the one hand, modulation of a single miRNA offers the opportunity to target multiple genes and regulatory networks simultaneously. However, for the same reason, caution and careful design are needed to prevent unwanted off-target effects.

Different modes of miRNA delivery are being pursued. Nuclease-mediated degradation before achieving target modulation is a major issue in achieving the desired outcome. Systemic administration of miRNA in a mouse model of hepatocellular carcinoma using adeno-associated virus resulted in inhibition of cancer cell proliferation, induction of tumor-specific apoptosis, and dramatic protection from disease progression without toxicity [42]. Single-stranded, cytoplasmic viruses of negative polarity capable of producing functional miRNAs have been described [43]. miRNA inhibitors termed miRNA sponges [44], antagomirs [45], locked-nucleic-acid-modified oligonucleotides [46], and reconstituted high-density lipoprotein nanoparticles [47] are some of the approaches that have been pursued. Currently recognized delivery barriers, development of novel nanomaterials, nanovector fabrication methods, and delivery approaches have been reviewed [48,49]. In yet another strategy, antibodies against various cell surface receptors were used for delivery; and upon intravenous administration, the complex was taken up by specific cells via receptor-mediated endocytosis [50].

Submicron vesicular structures that include exosomes and shedding vesicles are also being explored for delivery of exogenous therapeutic cargoes. One approach is based on engineering natural membrane vesicles loaded with a drug to target certain cell types. Characteristics of membrane vesicles are also being used in the design of nanoscaled drug delivery systems. Though there are challenges to overcome for successful clinical application, these approaches hold immense promise as novel drug carriers in the future In the second approach, essential characteristics of membrane vesicles are being used to design nanoscaled drug delivery systems [51]. Coupling engineered exosomes with nanotechnology is also the basis of immunotherapy leading to the development of cancer vaccines [52]. A better understanding of miRNA biology and their target genes, robust target binding, target tissue specificity, and the development of safe and effective delivery strategies can greatly enhance the therapeutic potential of miRNAs.

## 5. Diagnostic Tests

Some tests using miRNAs as biomarkers for clinical diagnosis are now available. The ProOnc TumorSource Dx is a laboratory test launched in 2009 by Prometheus Laboratories Inc (Los Angeles, CA, USA). It is used to identify the origin of metastatic cancer by determining the expression levels of 48 miRNA biomarkers in a sample of tissue taken from a tumor. The test can identify 25 different tumor types and can be used to detect cancer of unknown primary. Rosetta Genomics (Rehovot, Israel) offers three different tests designed to identify specific miRNA signatures. These tests can accurately identify the primary tumor site in metastatic cancer and cancer of unknown primary. Asuragen (Austin, TX, USA) has developed tests to diagnose pancreatic cancer. A number of excellent reviews on RNA interference therapeutics have been published recently [53–55] and hence are not addressed here.

## 6. Conclusions

The potential of using circulating miRNAs as biomarkers now extends beyond clinical oncology [8,56]. Advantages offered by biological markers vary depending on the therapeutic area. Whereas early detection is advantageous in oncology when there are no symptoms, the field of pain can benefit from being able to gauge pain and stratify a heterogeneous patient population [20]. miRNA expression profiles are more informative than mRNA expression profiles in a number of diseases [57]. With the successful detection of stable miRNAs in bodily fluids of humans and animals, the use of miRNAs as potential biomarkers is a reality. The functional relevance of the presence of stable miRNAs in blood is an area of active investigation.

Biomarkers can be used to determine the propensity to develop a disease, measure its progress, or predict prognosis [58]. Identifying informative biomarkers is an exceptionally valuable tool for evaluating clinical trial outcomes and for assisting physicians in choosing treatment options. A major problem hindering successful development of therapeutics is the mechanistic disconnect between preclinical and clinical studies. Evaluation of miRNA fingerprints in rodents that show a reversal of symptoms or pathological characteristics at efficacious doses of compounds under investigation can provide guidance for clinical trials. Efficacy trials could be conducted in mechanistically defined patient groups, guided by information obtained in preclinical and human volunteer models. In clinical trials, biomarkers can help in patient stratification, thereby increasing the chances of a successful outcome by targeting the appropriate population. In addition, biomarkers can pave the way to individualize treatment and thereby usher in a new era in personalized medicine [59]. Identification and development of biomarkers will play a major role in finding the right molecules, targets, and doses. The ability to identify several miRNAs as biomarkers rather than relying on one specific biomarker will increase the chances of successful treatment in a heterogeneous patient population. In addition, these studies could potentially determine if any of the miRNAs can be direct targets for future therapeutic interventions.

Interesting questions beyond the utility of miRNAs as biomarkers have been raised. Could the role of circulating miRNAs be to produce a systemic environment that is conducive to disease progression? Can we now overcome the technical challenges associated with delivery of oligonucleotides as therapeutic agents to the central nervous system using a serum-based exchange, either by supplementing downregulated miRNAs or by introducing antisense miRNAs for upregulated miRNAs using lipid-mediated delivery? Though much work lies ahead, circulating miRNAs are unequivocally ushering in a new era of novel biomarker discovery, intercellular communication mechanisms, and novel therapeutic intervention strategies. Improving the efficiency of miRNA delivery to achieve maximum efficacy in targeted cells with minimal toxicity will accelerate small RNA-based therapeutics. Understanding the mechanisms by which miRNAs are loaded into the exosomes, the secretory mechanism, its release into the bloodstream, the incorporation of extracellular miRNAs in mammalian cells, and its functional consequences are all research areas of immense interest. These discoveries will undoubtedly play an important role in the way we diagnose and treat diseases and will have a positive impact on human health.

## Appendix

**Biomarker** A biomarker or biological marker is any measurable substance in the body that can be an indicator of a normal biological or pathological state or serve as a measure of response to a therapeutic intervention.

**Epigenetic regulation** Epigenetics is defined as changes in gene expression and chromatin without accompanying changes in the DNA sequence [60]. Major epigenetic mechanisms include DNA methylation, covalent post-translational modifications of histone proteins, and RNA-mediated gene silencing.

**Next generation sequencing** Automated Sanger sequencing, commonly referred to as first generation sequencing technology, was used in the completion of the sequencing of the first human genome. However, its limitations, including throughput and cost combined with the need for large-scale comparative genomics studies in multiple organisms, resulted in the development of novel platforms for broader applications. These second-generation technologies, commonly referred to as next-generation sequencing [61], have revolutionized genome sequencing. Different next-generation sequencing platforms and their applications have been reviewed [62].
